# Estimating Copy Number and Allelic Variation at the Immunoglobulin Heavy Chain Locus Using Short Reads

**DOI:** 10.1371/journal.pcbi.1005117

**Published:** 2016-09-15

**Authors:** Shishi Luo, Jane A. Yu, Yun S. Song

**Affiliations:** 1 Computer Science Division, University of California, Berkeley, Berkeley, California, United States of America; 2 Department of Statistics, University of California, Berkeley, Berkeley, California, United States of America; 3 Departments of Mathematics and Biology, University of Pennsylvania, Philadelphia, Pennsylvania, United States of America; University of Texas at Austin, UNITED STATES

## Abstract

The study of genomic regions that contain gene copies and structural variation is a major challenge in modern genomics. Unlike variation involving single nucleotide changes, data on the variation of copy number is difficult to collect and few tools exist for analyzing the variation between individuals. The immunoglobulin heavy variable (IGHV) locus, which plays an integral role in the adaptive immune response, is an example of a complex genomic region that varies in gene copy number. Lack of standard methods to genotype this region prevents it from being included in association studies and is holding back the growing field of antibody repertoire analysis. Here we develop a method that takes short reads from high-throughput sequencing and outputs a genetic profile of the IGHV locus with the read coverage depth and a putative nucleotide sequence for each operationally defined gene cluster. Our operationally defined gene clusters aim to address a major challenge in studying the IGHV locus: the high sequence similarity between gene segments in different genomic locations. Tests on simulated data demonstrate that our approach can accurately determine the presence or absence of a gene cluster from reads as short as 70 bp. More detailed resolution on the copy number of gene clusters can be obtained from read coverage depth using longer reads (e.g., ≥ 100 bp). Detail at the nucleotide resolution of single copy genes (genes present in one copy per haplotype) can be determined with 250 bp reads. For IGHV genes with more than one copy, accurate nucleotide-resolution reconstruction is currently beyond the means of our approach. When applied to a family of European ancestry, our pipeline outputs genotypes that are consistent with the family pedigree, confirms existing multigene variants and suggests new copy number variants. This study paves the way for analyzing population-level patterns of variation in IGHV gene clusters in larger diverse datasets and for quantitatively handling regions of copy number variation in other structurally varying and complex loci.

## Author Summary

Regions of the human genome that vary in gene copy number are challenging to identify and analyze. This is particularly true for the immunoglobulin heavy variable locus (IGHV), which codes for a component of the antibody molecule. Previous approaches to interrogate the IGHV locus using locus-specific assays have provided detailed information about genetic variation, but tend to be low-throughput. Here, we introduce a method that leverages the increasing availability of large whole-genome sequencing datasets to genetically profile all functional IGHV genes in terms of a reference set of operationally defined gene clusters. We demonstrate this approach both on simulated data and on reads from a sixteen-member family of European descent. In the European family, not only did we find instances of known copy number variants, but also evidence of new variants. As larger, more diverse, datasets become available, our approach will allow the investigation of inter-individual copy number variation in larger samples for this and similarly hypervariable regions.

## Introduction

The variation between human genomes in gene copy number is understudied and poorly characterized. One such region where this variation is known to exist is the immunoglobulin heavy variable (IGHV) locus. It is a vital component of the adaptive immune system, containing the V genes that code for a component of the heavy chain of antibody molecules. Like other multigene receptor families, the gene segments in this region have been accumulated over time through a process of gene duplication and diversification [1–3]. As such, many of the genes in this locus are highly similar and there are repetitive DNA elements interspersed throughout the region. IGHV haplotypes (instances of the IGHV locus) vary not only by single nucleotide polymorphisms, but also in the copy number and ordering of gene segments [4–12]. All these characteristics make it difficult to study this region and, to date, only two reference sequences of the full IGHV locus exist [12, 13].

The human IGHV locus lies at the telomeric end of chromosome 14 and is approximately 1 Mb in length. In this 1 Mb region, there are about 40 functional genes, each approximately 300 bp in length. There are also approximately 80 non-functional pseudogenes in the region, so-called because they are either truncated or contain premature stop codons. Known allelic variants of individual IGHV genes are currently curated in the International Immunogenetics Information System (IMGT) Repertoire database [14]. Throughout this article, we suppress the standard prefix “IGHV” in gene names for ease of reading, e.g., we use 6-1 instead of IGHV6-1. The nomenclature for IGHV genes is further detailed in Materials and Methods.

Given the role of the IGHV locus in the adaptive immune response, IGHV genotypes are obvious candidates as genetic determinants for susceptibility to infectious disease. Several early targeted studies of the IGHV locus have implicated allelic variation and copy number in determining expressed antibodies repertoires and understanding disease susceptibility [5, 10, 11, 15–18]. Allele 3-23*03, for example, has been shown to be more effective in binding Haemophilus influenzae type (Hib) polysaccharide than the most common allele, 3-23*01 [19]. Despite such findings, however, the IGHV locus is rarely included in genome-wide association studies, due in large part to the lack of standard format and tools to quantitatively characterize variation in the region.

Lack of tools for genotyping the IGHV locus also hampers the burgeoning field of antibody repertoire sequencing [20–24], which is being used in numerous medical applications, including inferring the evolutionary path of broad and potent monoclonal antibodies against human immunodeficiency virus (HIV) [25–27], detecting blood cancers [28, 29], assessing the impact of aging on the antibody response [30], and measuring the adaptive immune response to vaccination [31, 32]. The first step in many of these studies is to align each read, sequenced from the antibody repertoire of an individual, to its germline gene. The current practice is to use germline alleles in a public database of all known alleles (such as the IMGT Repertoire database) for alignment. Aligning to all germline alleles is a severe limitation of the process because after undergoing somatic hypermutation, antibody sequences may be so different from the germline that the top-matching allele in the database no longer corresponds to the germline allele in the individual.

The increasing availability of whole-genome sequencing (WGS) provides a new opportunity to investigate genetic variation in the IGHV locus. Specifically, the large sample sizes of these WGS datasets and the high-throughput manner in which the data can be analyzed could provide valuable information. This approach can complement and guide genotyping efforts based on locus-specific assays [9–12]. Yet there are currently no methods to interrogate the IGHV locus using WGS short reads (though a tool that extracts genotypes from long contigs exists [33]).

Here, we address this pressing need for methods that quantitatively characterize the IGHV locus from WGS short-read data. By leveraging the IMGT database of known alleles, we construct a pipeline that gives a systematic description of the IGHV locus from short-read data. This description is in terms of a set of operationally defined gene clusters, so called because each cluster comprises IGHV alleles that are operationally indistinguishable. Our method does not attempt to reconstruct the organization of the locus or sequence the intergenic regions, both of which are important and challenging tasks. It does, however, allow the quantification of coarser measures of genetic variation. With reads as short as 70 bp and with coverage of 30×, our pipeline accurately detects the presence of gene clusters from simulated reads of the two known IGHV reference sequences (GRCh37 and GRCh38). With sufficiently long read lengths (250 bp), the pipeline also outputs accurate nucleotide sequences of gene segments present in single copy. We then run the pipeline on an empirical dataset of whole-genome sequencing reads from a sixteen member family, obtaining for the first time distributions of copy number in this family. Our copy number calls are consistent with the family pedigree and confirm known multigene variants of the IGHV locus. Our results also suggest evidence of copy number variants that are mosaics of the existing reference haplotypes and variants that might be transitional between them.

## Results

### Hierarchical clustering to define operational gene clusters

**Fig 1:**
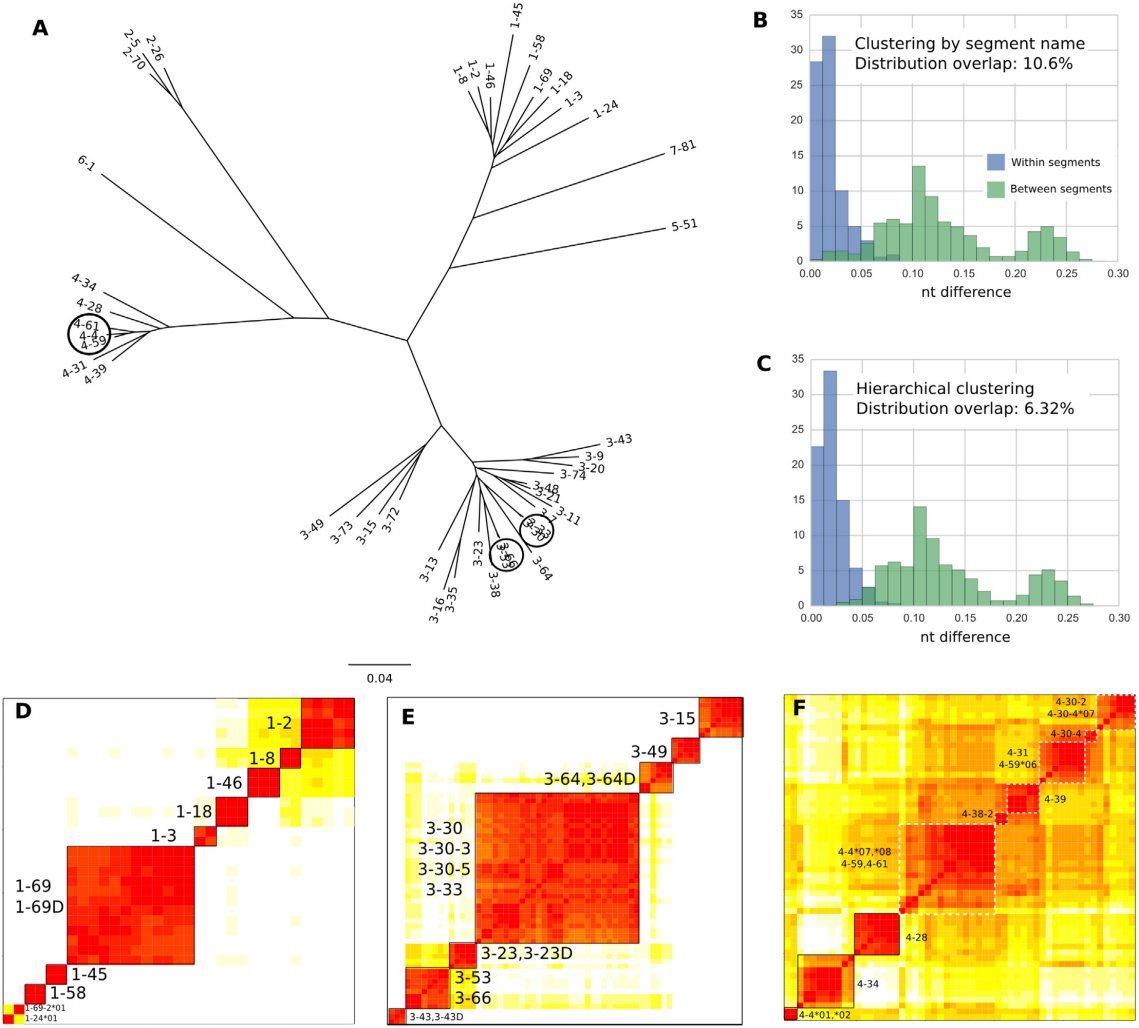
Alleles clustered according to nucleotide similarity. (A) Phylogenetic tree reconstruction of the gene segments in the haplotype sequenced in [13] (which is the GRCh37 haplotype). Circles highlight alleles that are evolutionarily very close. Tree made using neighbor-joining method in ape package in R based on Hamming distance between multiple sequence alignment. (Phylogenetic reconstruction using BEAST [35] led to a qualitatively similar tree). Allele numbers are suppressed for clarity. (B) Distribution of percent nucleotide difference (Hamming distance divided by alignment length) between alleles from same IMGT segment (blue) compared against alleles from different segments (green). Alleles from duplicate segments (e.g. 1-69 and 1-69D) have been merged for this analysis. (C) Same as (B) but with alleles partitioned by operationally defined gene clusters rather than IMGT segment name. (D-F) Heatmaps of matrices of Hamming distance between alleles. Rows and columns are ordered according to gene clusters found by hierarchical clustering as described in Materials and Methods. Color spectrum ranges linearly from red to white for nucleotide distances 0-10%. Differences greater than 10% are white. (D) Alleles from family 1. (E) Alleles from family 3. Full set of alleles in S3 Fig. (F) Alleles in family 4. Dashed white squares indicate possible gene clusters.

The main difficulty in accurately genotyping the IGHV locus is the high level of similarity between alleles of different gene segments. Fig 1A illustrates the level of nucleotide similarity between the IGHV segments in GRCh37. For example, the alleles of segments 3-30 and 3-33 in GRCh37, circled in Fig 1A, differ in only 1.4% of their nucleotides. Since some segments have alleles that differ by more than 1.4% in their base pairs (Fig 1B), it becomes problematic to distinguish between alleles of the GRCh37 genes 3-30 and 3-33 based on nucleotide dissimilarity. To be more concrete, if one had reads of length 100 bp from a haplotype containing both 3-30 and 3-33 segments, it would be algorithmically very difficult, if not impossible, to correctly map reads that are from regions common to 3-30 and 3-33. We note that this difficulty in distinguishing between alleles becomes even more pronounced when analyzing antibody repertoire sequencing data, where somatic hypermutation further confounds the matching of repertoire sequences to germline alleles [34].

This problem also occurs with other gene segments: across all full-length functional IMGT alleles, there is a 10.6% overlap in the distribution of nucleotide differences between alleles with the same segment name and alleles with distinct segment names (Fig 1B). Reads from the alleles in this overlapping region cannot be operationally distinguished from each other, leading to unreliable and ambiguous read mapping results. Thus, in the context of mapping short reads, it does not make sense to keep these alleles separate, so we pool them together into units we call “operationally defined gene clusters”, or gene clusters for short. As we show in the next sections, this strategy allows us to extract useful information, such as copy number estimates, with less ambiguity.

To determine these gene clusters in a systematic manner, we perform hierarchical clustering within each family of full-length, functional IMGT alleles (Materials and Methods). By grouping the alleles together according to their sequence similarity, we reduce the overlap to a greater extent than grouping according to segment name alone. (Fig 1C). We see that although we cannot eliminate the overlap completely, in most gene clusters, alleles are within 5% nucleotide differences of each other.

Some families have clearly defined gene clusters. In family 1, the gene clusters correspond to segment name, as long as duplicate segments 1-69D and 1-69 are merged (Fig 1D). In families 2 and 5, which have three and two segments respectively, the alleles cluster by segment name (S1 and S2 Figs). In family 3, six segments that have distinct names—namely, 3-30, 3-30-3, 3-30-5, 3-33, 3-53, and 3-66—form two gene clusters {3-30, 3-30-3, 3-30-5, 3-33} and {3-53, 3-66} (Fig 1E). Families 6 and 7 each have only one functional gene segment and therefore do not require clustering.

Surprisingly, the same clustering algorithm that leads to clean gene clusters in the other families fails to identify clear-cut gene clusters in family 4 (Fig 1F). Not only are the boundaries between gene clusters fuzzy in this case, but alleles of the same segment cluster separately. For example, 4-4*01 and 4-4*02 cluster separately from 4-4*07 and 4-4*08. The alleles in family 4 also seem to be more similar to each other than alleles in other families. It is not clear why alleles in family 4 in particular should cluster poorly compared to those of other families. Gene conversion events in IGHV family 4 and a more recent common ancestor than that of other IGHV families are both possible explanations that are consistent with the observed distance matrix. A better clustering, based on a combination of mutational distance and indel distance, was ultimately used to define the gene clusters for family 4 (S4 Fig).

With the caveat that family 4 gene clusters are more speculative, Table 1 summarizes the operationally defined gene clusters as determined by hierarchical clustering. Only gene clusters which disagree with the IMGT V gene segment name are listed. For the remainder of this manuscript, we use the term *gene cluster* to refer to our operationally defined clusters and IMGT V gene segment to refer to the standard IMGT nomenclature. When an operationally defined gene cluster is the same as an IMGT V gene segment—e.g., in the case of segments in gene families 5, 6, and 7—we use the terms interchangeably. It may help the reader to keep in mind that with the exception of family 4, the majority of gene clusters coincide either with the IMGT V gene segment names, or with IMGT V gene segments merged with their duplicates (e.g., 3-64 and 3-64D).

**Table 1:** Operationally defined gene clusters from our hierarchical clustering analysis. Only those that differ from the current IMGT nomenclature are listed. That is, gene clusters that agree with the IMGT V gene segment, such as 6-1, are not shown.

Gene cluster name	Alleles, under current IMGT nomenclature
1-69	All 1-69 and 1-69D alleles
2-70	All 2-70 and 2-70D alleles
3-23	All 3-23 and 3-23D alleles
3-30	All 3-30, 3-30-3, 3-30-5 and 3-33 alleles
3-43	All 3-43 and 3-43D alleles
3-53	All 3-53 and 3-66 alleles
3-64	All 3-64 and 3-64D alleles
4-4*01	4-4*01, 4-4*02
4-30-2	All 4-30-2 alleles and 4-30-4*07
4-31	4-30-4*01, 4-30-4*02, 4-31*01-*04, 4-31*10
4-31*05	4-31*05
4-59	4-4*07, 4-4*08, and all 4-59 alleles
4-61	4-61*01, 4-61*03-*05, 4-61*08
4-61*02	4-61*02

### Pipeline performance on simulated reads

**Fig 2:**
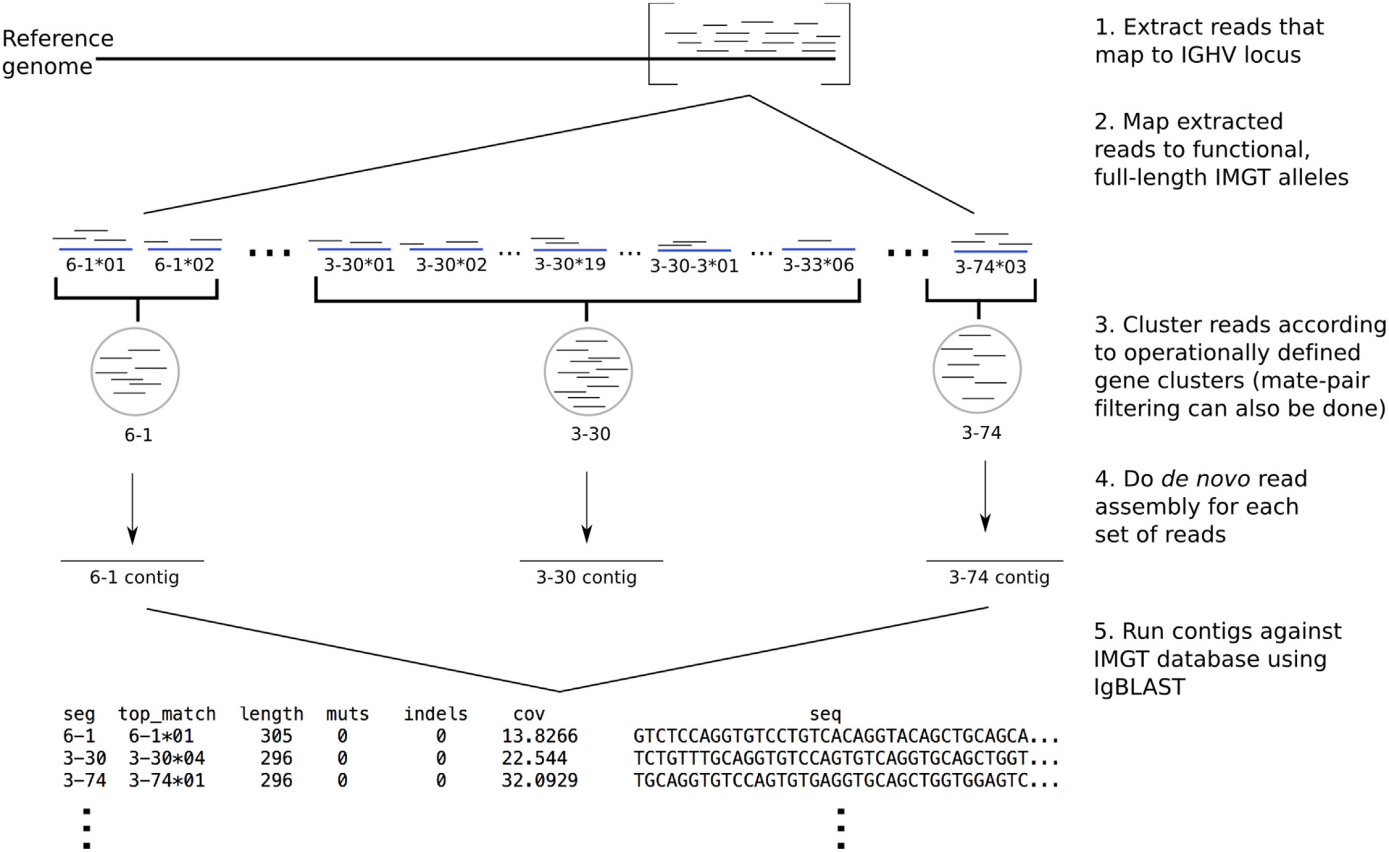
Schematic of genotyping pipeline. 1. WGS reads (short thin black horizontal lines) that map to the IGHV locus of a single individual are extracted from full set of reads. 2. These extracted reads are mapped using Bowtie2 [36] to known functional V gene segment alleles (thick blue horizontal lines) curated from the IMGT database [37]. 3. Mapped reads are pooled according to the gene clusters described in the Results section. At this stage, extra filtering steps using additional information like mate-pair data can also be applied. 4. Local assembly using SPAdes [38] is performed on reads to produce contigs (long thin black horizontal lines) corresponding to each gene cluster. 5. The resulting contigs are identified using stand-alone IgBLAST [39]. The final output contains, for each individual and each assembled contig: the closest-matching existing allele, the length of match, the number of nucleotide mutations or indels that separate the contig from the closest-matching allele, the read coverage of the contig as reported by SPAdes, and the nucleotide sequence of the contig.

**Fig 3:**
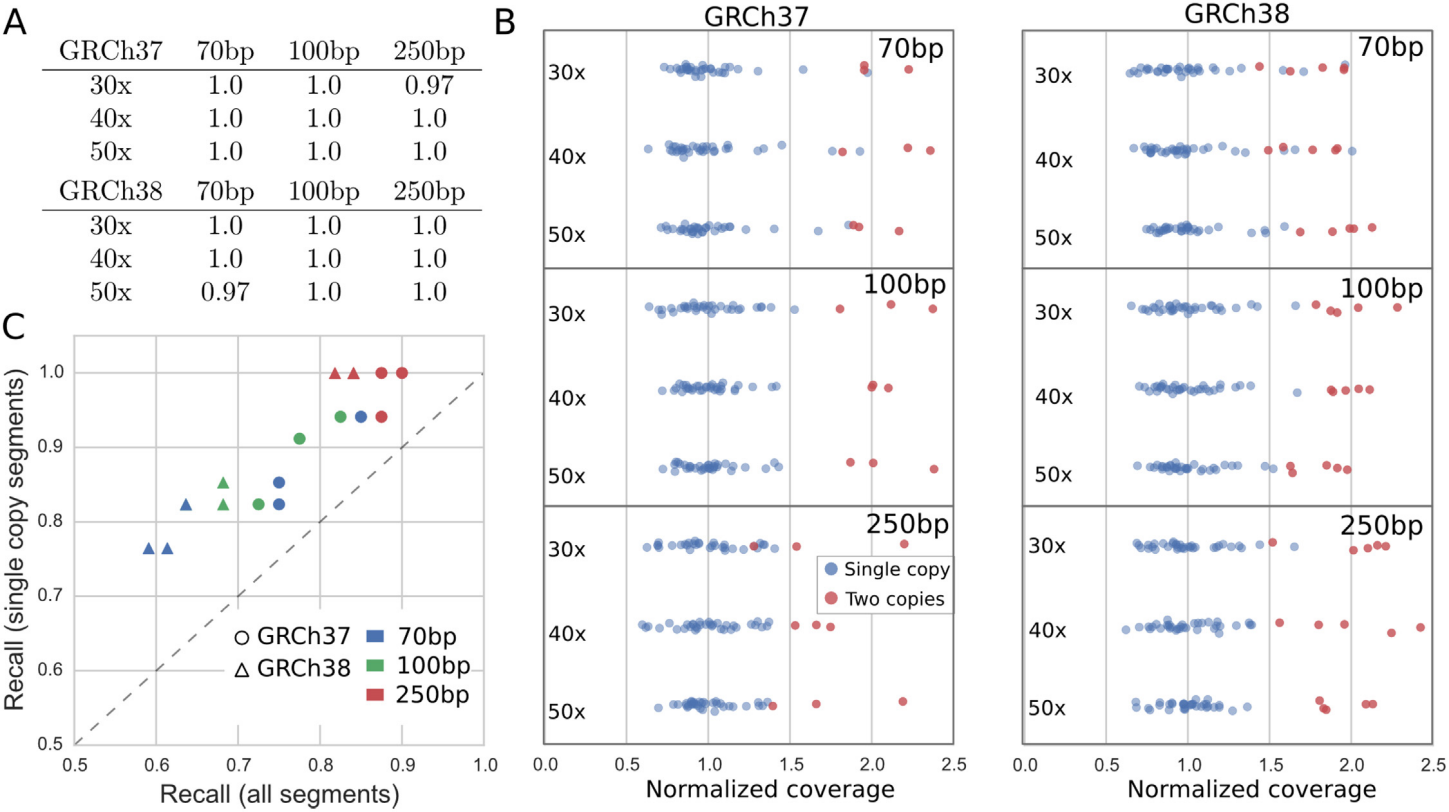
Performance of pipeline on simulated reads from GRCh37 and GRCh38 for varying coverage depths and read lengths. (A) Recall fractions of the pipeline for the two human reference genomes (precision fractions are all 1 and not shown). Recall is calculated as the fraction of gene clusters in the reference genome that are correctly called by the pipeline. (B) Read coverage depth of each assembled gene cluster (the point estimate for copy number) colored by actual copy number (detailed in S2 Table) in the reference genome. Raw read coverage depth has been normalized by the average coverage of single-copy segments. Jitter has been applied to the vertical coordinates to better show their distribution. (C) The recall of alleles for all gene clusters versus the recall for gene clusters known to be present in the reference in single copy. Each point is one reference genome, coverage depth, read length combination. Note that two red triangles overlap at the point (0.84, 1.0). Different coverage depths are not indicated because there is no pattern between coverage depth and allele reconstruction accuracy.

The operationally defined gene clusters (Table 1) address the main difficulty in genotyping the IGHV locus and is the key idea behind our data pipeline (Fig 2). Without this crucial step, it is difficult to determine IGHV alleles from read mapping alone (S1 Table). The input of the pipeline is a file of whole-genome sequencing reads from an individual. The output is a genetic profile of the IGHV locus: for each gene cluster it reports a point estimate of copy number, the closest matching existing IMGT allele, and a nucleotide sequence of the contig assembled from reads mapping to the gene cluster. Fig 3 shows the performance of our pipeline at three levels of genotypic resolution on simulated reads from the two complete IGHV haplotype sequences (Materials and Methods).

At the coarsest scale, we ask whether the pipeline correctly identifies the presence or absence of each gene cluster. We find the pipeline to be highly accurate, with precision of 100% for all coverage depth (30×, 40×, 50×) and read length (70 bp, 100 bp, 250 bp) combinations. This means that all the gene clusters identified by our pipeline are present in the reference. The recall, the fraction of gene clusters in the reference that are identified by our pipeline, is 100% for all but two of the coverage depth/read length combinations (Fig 3A).

At the next level of resolution, we ask whether the pipeline can correctly determine the copy number of each gene cluster. We use the read coverage depth of the assembled contig as our point estimate for copy number. Fig 3B shows that contig coverage depth is indeed correlated with copy number, though there is variation above and below the true copy number and some gene clusters which are present in single copy have high coverage depth. This is because pseudogenes in the IGHV locus, which are not included in our reference set, may share common subsequences with functional genes. Reads from pseudogenes can therefore be erroneously mapped, artificially inflating the contig coverage depth. This is particularly an issue with 70 bp length reads as these reads are more likely to completely fall within a conserved region. This problem can be partly alleviated with paired-end reads, a strategy we use on the real dataset in the next section.

At the highest level of resolution, we compare the assembled contig obtained from the pipeline to the known nucleotide sequence for each gene cluster. When a gene cluster is only present in single copy in the locus, and if the read lengths are 250 bp, the recall of the nucleotide sequence is 100% in all but one of the simulated datasets (Fig 3C). With shorter reads, the frequency of correctly calling alleles is lower. As with copy number determination, this lower accuracy is likely due to erroneously mapped reads from pseudogenes and highly similar functional genes that interfere with the assembly algorithm. For the same reason, when a gene cluster is present in more than one copy and as different alleles, the allele calls are also less accurate. Note that higher coverage depth does not necessarily improve accuracy because the error arises not from sequencing error, which occurs in random locations and can be mitigated with higher coverage depth, but from erroneously mapped reads, which are systematically incorrect regardless of coverage.

### Genotyping the Platinum Genomes dataset

We next apply the pipeline to the publicly available Platinum Genomes dataset [40], a set of whole-genome sequencing reads of length 100 bp at roughly 30× coverage depth from a family of 16 individuals (four grandparents, a mother, a father, and ten children, all of European ancestry). Because these reads are paired, we perform an additional filtering step (Materials and Methods) to discard reads that are potentially from pseudogenes in order to improve our allele calls and decrease the false discovery of duplicated genes.

**Fig 4:**
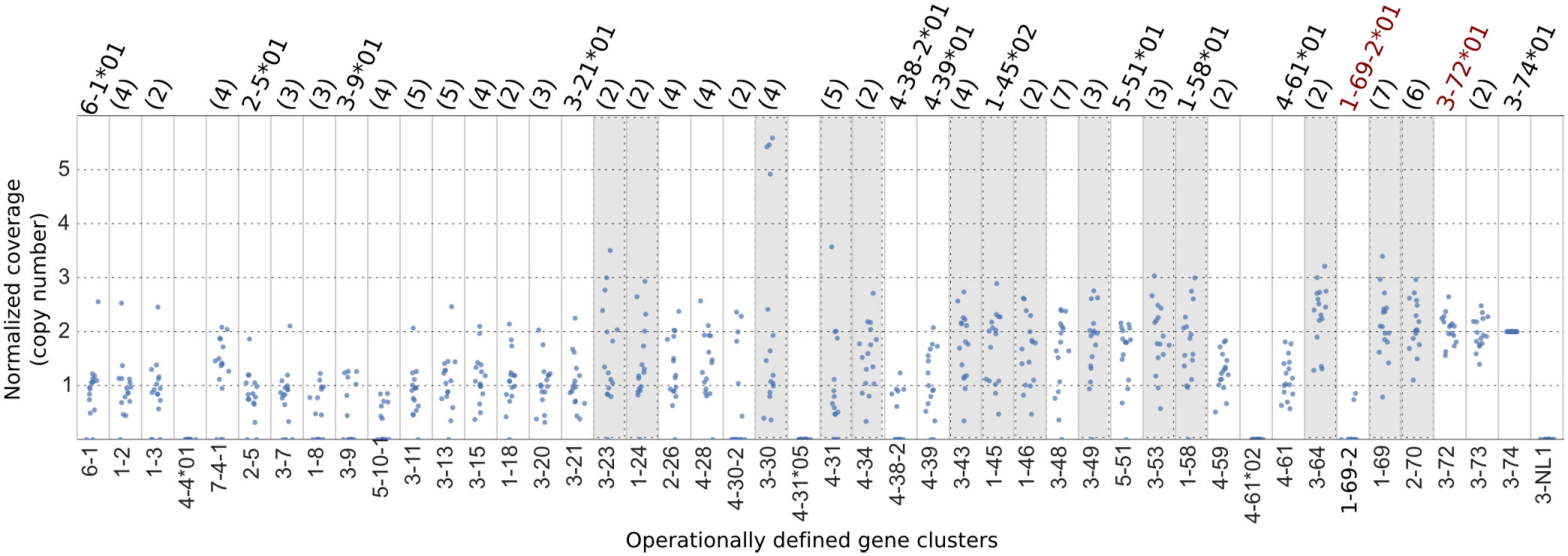
Dotplots of estimated copy number for each gene cluster for Platinum Genomes data. The Y axis is the normalized coverage, i.e. the coverage depth divided by half of the coverage depth of segment 3-74 (assumed to have two copies). On the X axis, gene clusters are ordered, where possible, according to their location in the reference genome, from 6-1 (centromeric end) to 3-74 (telomeric end). The number in parentheses above each gene cluster is the number of unique allele sequences found in the Platinum Genomes family. If only one allele was found, its name is given (in the case where a segment has only one known allele in the IMGT database, the allele name is in red). Shaded columns indicate gene clusters that likely have more than one copy per chromosome. The outliers for gene clusters 6-1, 1-2, and 1-3 all correspond to a single individual, NA12891, who had relatively uniform coverage across the locus (S5 Fig). Horizontal jitter has been applied to all points to better illustrate the distribution.

A summary of copy number and allelic variation in IGHV gene clusters in this dataset is shown in Fig 4 (S3 Table lists all raw coverage depth values from the dataset). For all the results that follow, the raw coverage depth of each gene cluster is scaled by the coverage depth of segment 3-74 in the same individual to eliminate variation due to differences in read coverage between individuals (IMGT V gene segment 3-74 coincides with the gene cluster 3-74). We choose segment 3-74 because it has no documented examples of copy number variation and is located at the telemoric end of the chromosome. Specifically we assume that 3-74 has two copies, one on each chromosome, and divide the coverage depth of all other gene clusters by half of the coverage depth of 3-74. A normalized coverage depth of 1 therefore corresponds to a single copy on one, but not both, of the chromosomes. Note that the coverage depth tends to decrease towards the 6-1 end of the locus due to VDJ recombination, an issue we will return to in the Discussion.

#### General patterns of variation

Fig 4 shows for the first time the distribution of copy number variation in a sample of individuals, across all IGHV gene clusters. Variation exists in copy number within and between gene clusters. Some, such as 3-72 and 3-73 are present in all individuals as two copies, one on each chromosome. Others, such as 1-8, 3-9, 5-10-1, 4-38-2, and 1-69-2, are either absent (coverage of zero) or present as a single copy on one chromosome. We note that even though the Platinum Genome reads were mapped to GRCh37, we are nevertheless able to assemble full nucleotide sequences of 4-38-2 and 1-69-2 that are not in the reference and which are quite distinct from all other alleles. Normalized coverage around the value of three or higher indicates a segment has a duplicate on the same chromosome (segments shaded in grey in Fig 4). These include gene clusters 3-23, 3-30, 4-31, 3-43, 1-46, 3-53, 3-64, 1-69, and 2-70, which are known to have duplicates, but also 1-24, 4-34, 1-45, 3-49, and 1-58, for which duplicates have not been previously documented. These latter gene segments are new candidates for copy number variants and topics for further study.

Thirteen out of the forty two gene clusters (about 30%) found in the family are each represented by the same single allele in all sixteen members of the family. The unique alleles corresponding to these clusters are denoted in Fig 4 along the top of the plot. This strongly suggests that these clusters are homozygous in the four unrelated grandparents. Genotyping of a larger sample will ascertain whether this set of common alleles is shared by all individuals of European ancestry or is a by-product of our sample being a small set of closely related individuals. In either case, our pipeline and approach begins to address the question of whether a subpopulation can be uniquely identified by a common set of IGHV alleles.

#### Multigene copy number variants

**Fig 5:**
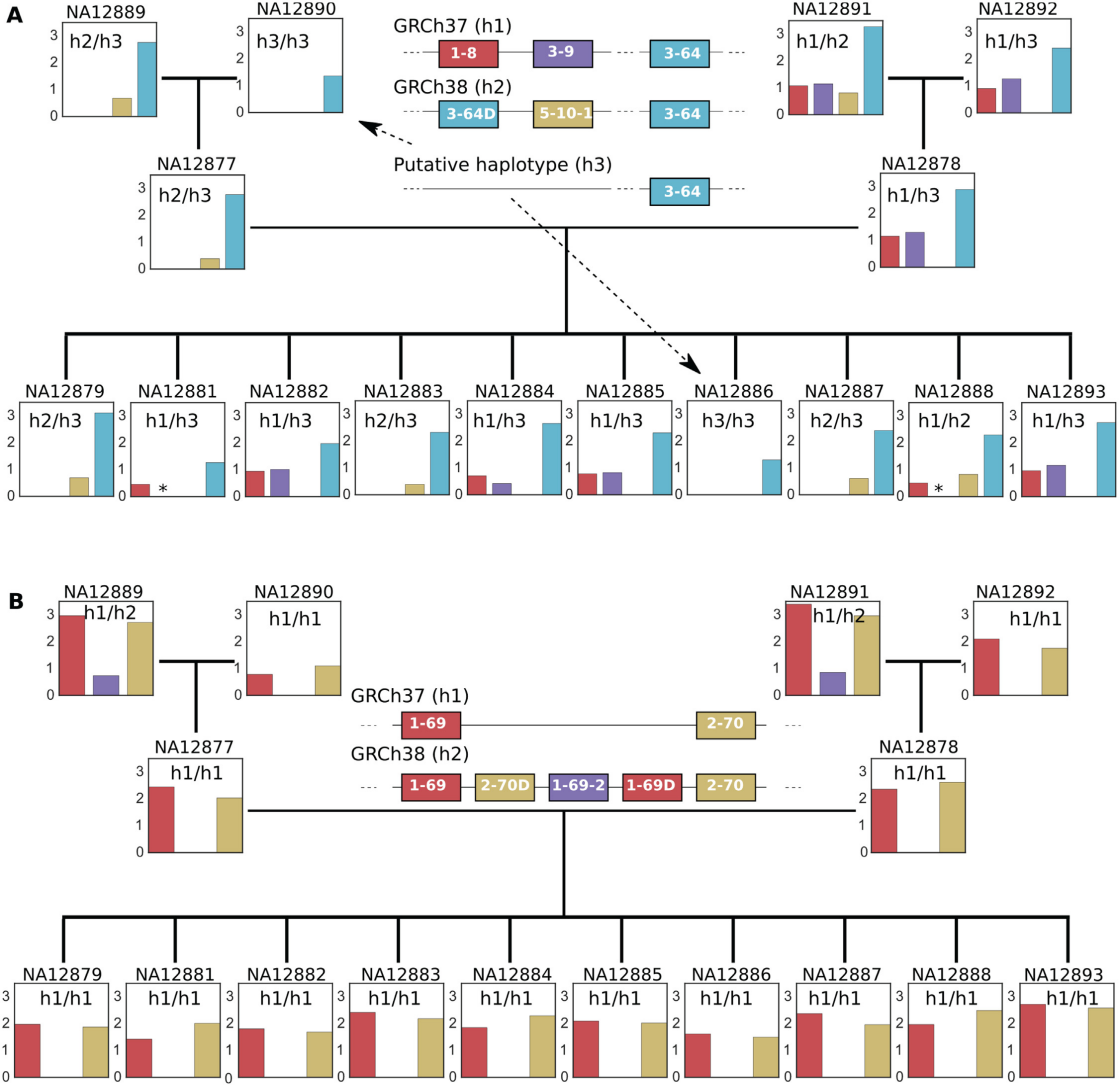
Coverage calls for two multigene copy number variants in GRCh37 and GRCh38. (A) Alternative haplotypes 1-8/3-9 and 3-64D/5-10-1. Additional manual examination of pipeline output shows, consistent with the putative haplotypes in each individual, that 3-9 is present in NA12881 and NA12888 (indicated by stars ‘*’) and that 5-10-1 is not present in NA12886. (B) The insertion haplotype 2-70D/1-69-2/1-69D. In both subfigures, Y axis on each bar plot is normalized coverage. Each bar is colored according to the segment or gene cluster it corresponds to. The putative haplotypes for each individual are indicated on the coverage bar plot. Individuals are arranged according to their family tree. NA12877 and NA12878 are the father and mother respectively.

We next looked for the presence in family members of two copy number variants that differ between the GRCh37 and GRCh38 reference haplotypes and that involve more than one gene segment or cluster (Fig 5). Using knowledge of the family pedigree, we reconstruct putative haplotypes for these variants in each individual.

In the case of alternative haplotypes 1-8/3-9 (GRCh37, indicated in Fig 5A as ‘h1’) and 3-64D/5-10-1 (GRCh38, indicated as ‘h2’), our point estimates for copy number show that maternal grandparent NA12891 carries both configurations, one on each chromosome. In contrast, the 1-8/3-9 type is entirely absent from the paternal side of the family (Fig 5A). We manually checked our pipeline output to verify that the copy number calls in the children are consistent with the pedigree. Indeed, both NA12881 and NA12888 which appear to be missing the 3-9 segment, generated reads that mapped to a full-length 3-9*01 allele (indicated by ‘*’ in plots). This is consistent with NA12881 carrying the GRCh37, but not GRCh38, configuration and NA12888 carrying both the GRCh37 and GRCh38 configurations. (Our automated pipeline did not call the 3-9 segment because the coverage of that segment was too low for the assembler to run). We also verified that NA12890 and NA12886 do not carry the 5-10-1 segment, suggesting a new haplotype (h3), different from GRCh37 and GRCh38, which contains a single 3-64 segment without either the 1-8/3-9 or 3-64D/5-10-1 gene combinations. In both these individuals, there were allele calls and positive coverage for genes towards the 6-1 end of the locus, indicating that the absence of 1-8/3-9 and 3-64D/5-10-1 genes are not due to VDJ recombination in the cell type or low coverage (S6 Fig). Further, these two individuals appear to be homozygous for this new haplotype, suggesting that the haplotype is common.

Another multigene copy number variant, the 2-70D/1-69-2/1-69D insertion, is carried by two grandparents (NA12889 and NA12891) on one chromosome but not the other (Fig 5B). The insertion did not transmit to the parents or children, with neither the presence of 1-69-2 nor elevated coverage for 1-69 and 2-70 present in those individuals.

Interestingly, although all the children are homozygous for the GRCh37 (1-69/2-70) haplotype without the insertion, three of them have the GRCh38 (3-64D/5-10-1) haplotype on at least one chromosome. This implies that there are IGHV haplotypes different from both reference genomes and that these haplotypes are possibly mosaics of the reference genomes. Analysis of these two variants therefore not only confirms their presence in the Platinum Genomes sample, but also demonstrates that different configurations are present in the same ethnic population and suggests that many more configurations may exist. This is in line with resequencing efforts that have discovered novel sequences not found in GRCh37 [41–44], and also IGHV-specific studies [4, 9–11] that feature variants different from those in the two reference genomes.

#### High copy number variation in the operationally defined 3-30 gene cluster

Among all the gene clusters, 3-30 exhibited the most variation in coverage depth (Fig 4). This confirms previous findings that the V gene segments in this cluster—3-30, 3-30-3, 3-30-5, and 3-33—often exhibit differences in copy number [4, 7, 11, 12]. With our pipeline we now are able to begin collecting previously unknown quantities, such as the mean and range of copy number of the gene cluster comprising these highly similar segments. This information will also help determine whether copy number is segregating in different subpopulations.

**Fig 6:**
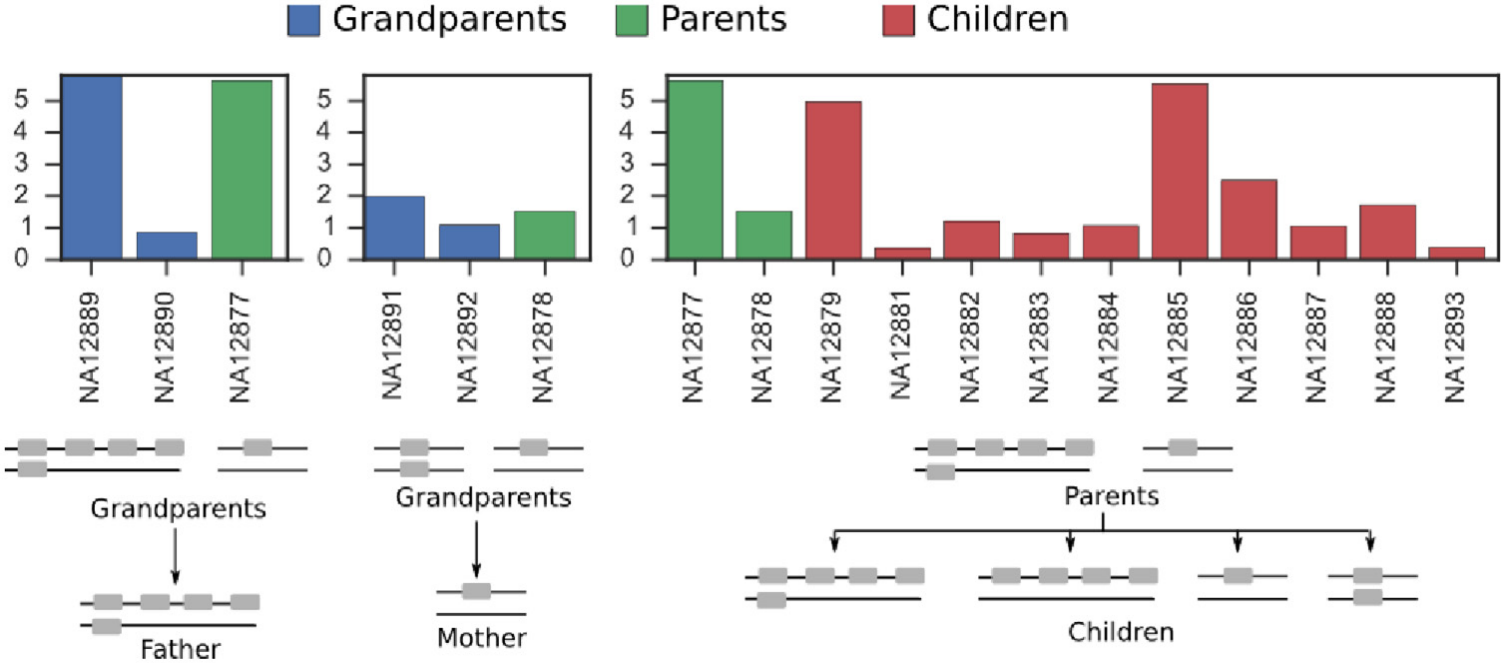
Copy number variation in the 3-30 gene cluster. Below each bar chart of coverage values is a putative reconstruction of the copy number on each chromosome for each individual in the family. Y axis is normalized coverage.

Using pedigree information as a constraint, we reconstructed the putative copy number of gene cluster 3-30 on each chromosome in each member of the family (Fig 6). Its abundance ranges from zero to four copies on a chromosome. To our knowledge, previous results about the variation in copy number of this segment were not linked to the ethnicity of the individual. Since our subjects are all of European ancestry, this may be the first result about copy number variation within a single ethnic group for the gene cluster 3-30.

#### New 7-4-1 allele

With the exception of 7-4-1, we found exact matches to IMGT alleles among all the segments in the Platinum Genomes dataset. In the case of 7-4-1, none of the assembled alleles exactly matched an existing IMGT 7-4-1 allele. To eliminate the possibility that the allele calls were confounded by reads from known pseudogenes, these alleles were determined after applying an extra filtering step to reads that mapped to the 7-4-1 segment (Materials and Methods). One of these alleles was five nucleotide mutations away from 7-4-1*04 and present in five individuals (S7 Fig shows an alignment of the new allele with 7-4-1*04). Our finding of an allele that is not in the IMGT database is in line with recent reports of novel alleles found using antibody repertoire sequences [8, 45–47].

## Discussion

With the approach introduced here, we can begin to obtain population-level statistics on the IGHV locus from WGS data and systematically quantify variation with respect to operationally defined gene clusters. Given the small sample size of the Platinum Genomes data, we have focused here on quantifying variation in genes known to vary in copy number. As larger whole-genome sequencing datasets become available, it will be possible to compare IGHV copy number profiles at the population scale. These profiles can then be studied to find correlations between multiple gene segments and clusters and to discover new copy number variants. Even with the coarse measure of presence/absence of gene clusters, we can begin to address basic open questions such as whether there is a minimal number of IGHV gene clusters required for a healthy immune system and whether there is a common core set of IGHV gene clusters that are shared by all individuals.

**Fig 7:**
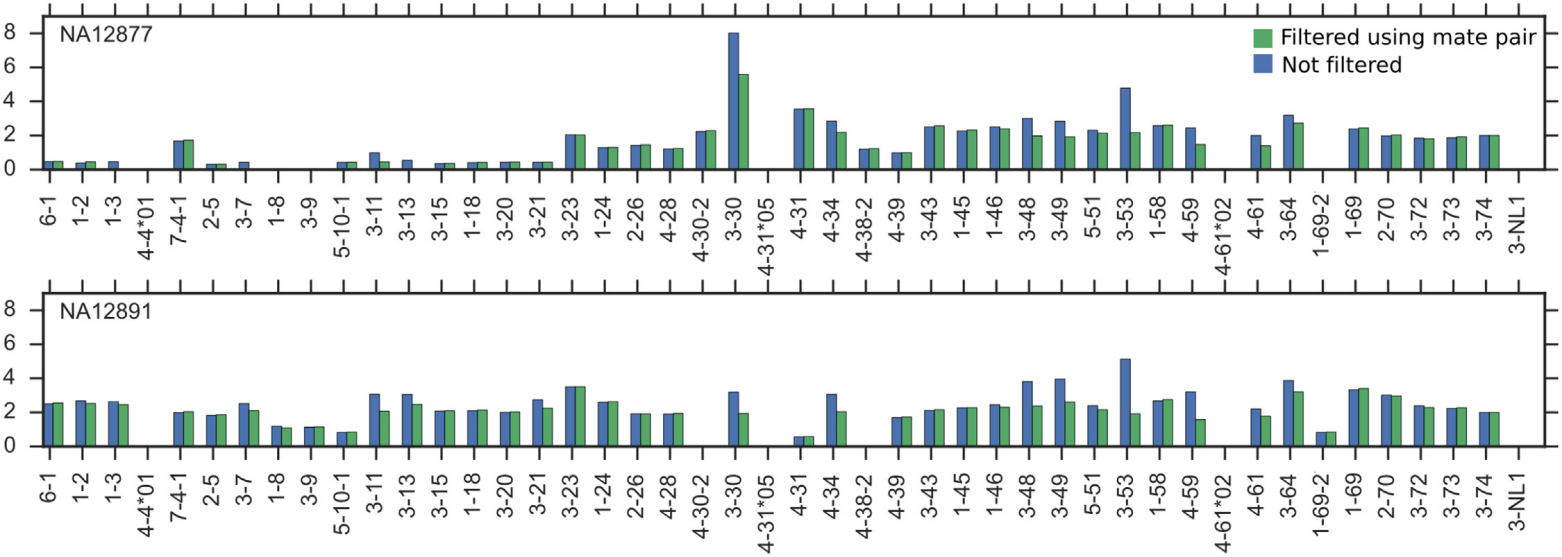
Complications arising from cell type and diploidy in Platinum Genomes dataset. Example of two individuals that differ in the uniformity of coverage over gene clusters. Y axis is normalized coverage.

Our study makes clear that read depth information can be used to accurately determine the presence and absence of gene segments and clusters. However, complications remain for ascertaining copy number and allelic content to high accuracy. The first complication arises from the cell type on which whole-genome sequencing is commonly performed. The Platinum Genomes data were generated from immortalized B lymphocytes. The IGHV locus in these cell types have undergone VDJ recombination. This rearrangement, which truncates the IGHV locus, confounds the correlation between read coverage depth and copy number of a gene cluster. We can see this from the pipeline output, where coverage depth tends to decrease towards the centromeric end of the locus. The extent of this decrease can be quite marked, for example in the case of NA12877, or not noticeable at all, for example in NA12891 (Fig 7; the distribution of read coverage depth of all the individuals is summarized in S5 Fig). If one knew the number of B cell lineages used to prepare the library and the fraction of haplotypes that underwent rearrangement, it is possible to adjust the raw coverage values to reflect actual coverage values (S1 Appendix). However, in the case of the Platinum Genomes data, this information is unavailable. As whole-genome sequencing becomes more widespread, we anticipate that datasets from other cell types will become available and this issue will be resolved.

The second complication is that the majority of whole-genome sequence reads are generated from diploid cells. Because the majority of segments on both chromosomes are of different alleles, the single allele call generated by our pipeline may be composed of sequence from all the alleles present or represent just one of the alleles. Allele calls can thus hide the heterozygous state of an individual. S8 Fig gives examples of segments which are present as two alleles in the family and for which the allele calls are misleading. This problem could be addressed with an assembler or method tailored to reconstruct the nucleotide sequence of alleles of short genomic regions (popular assemblers are currently designed for whole-genome assembly). Such a task is nontrivial, however, and beyond the scope of the current paper. There has been some success in identifying unique alleles using an alternative data type: antibody repertoire sequencing data [8, 16, 46]. However, such studies cannot directly quantify the copy number of an exactly duplicated gene because read abundances in these studies are not correlated with germline gene abundances due to differential gene usage in the development of an antibody repertoire. Furthermore, the V gene segment can be truncated during the genomic rearrangement for producing the antibody coding sequence, so that full-length alleles may not always be obtained from antibody repertoire sequencing data.

We note that there are many existing methods for estimating copy number based on coverage depth using whole-genome sequencing [48–52]. These methods, however, do not utilize the IMGT database of IGHV alleles nor do they specifically target the IGHV locus, a region with a higher amount of repetitions and duplications than most of the genome. They therefore may be prone to biases introduced by targeting the entire genome, which has loci of varying characteristics, rather than targeting a particular region. Additionally, some existing methods [53] intended for whole exome sequencing may be further biased when introduced to data from whole-genome sequencing.

True determination of IGHV haplotypes must ultimately come from sequencing the 1 Mb region in its entirety and in multiple individuals. Indeed, because the GRCh37 reference is a chimera of three diploid haplotypes [13], there is currently only one true reference haplotype for the IGHV locus. However, the technology to accurately sequence structurally varying regions remains expensive and low-throughput. We can instead take advantage of the increasing availability of whole-genome sequencing datasets and the extensive IMGT database to systematically describe this locus in a high-throughput manner albeit at lower genotypic resolution. Using this strategy, we have found evidence of haplotypes that are mosaics of reference genome configurations or that are transitional between them. The existence of these haplotypes further indicates that our approach of representing the locus in terms of a reference set of gene clusters is a less cumbersome means of cataloging the high copy number variation in this locus, compared to reconstructing full sequences of the IGHV locus with annotated breakpoints.

The fundamental strategy applied here is not specific to the IGHV locus. Reads from whole-genome sequencing datasets can similarly be used to characterize other gene families and in other species, where the genes are of comparable length and similar level of diversity. Some examples include T cell receptor genes and olfactory receptor genes. The use of whole-genome sequencing data therefore need not be restricted to single nucleotide variants, but can also be applied to study regions exhibiting copy number variation.

## Materials and Methods

### The standard naming convention for IGHV genes

IGHV genes are named according to their “family” and genomic location. The families, numbered 1 to 7, comprise genetically similar genes. The segment 6-1, for example, is in IGHV family 6 and is the first gene in the locus, counting from the centromeric end. Gene names with a suffix “D” denote a duplicate gene, for example 1-69D, while an appended number, for example 1-69-2, indicates that the gene was discovered subsequent to the original labeling and is located between 1-69 and 2-70. An allelic variant of an IGHV gene is denoted by a *01, *02, etc., as in 1-69*01, 1-69*02.

### Hierarchical clustering

Nucleotide sequences for IGHV gene alleles were downloaded from the IMGT database [37]. Only full-length functional alleles were used for clustering. Multiple sequence alignment was performed on each family of alleles using Fast Statistical Alignment with default parameterization (FSA, [54]). The aligned alleles were then clustered using the hclust function in R [55] (method parameter set to “single”, although using the “complete” method gives the same result for all families with the exception of family 4). The clustering algorithm tries to organize the alleles so that alleles with higher nucleotide similarity are in the same cluster while those with lower nucleotide similarity are in different clusters. The algorithm starts by first putting each allele in a separate cluster, then iteratively joining the two most similar clusters. For example, the cladogram in S3 Fig shows how the clusters are formed for family 3 alleles. For all the IGHV families except family 4, gene clusters were determined using distance matrices calculated from Hamming distance based on FSA alignment, with gap differences treated in the same way as mutations. Visual inspection of the alignment of family 4 suggested that indels may be important in partitioning the alleles. Hence, a combination of an evolutionary distance “TN93” (based on [56]) and indel distance (number of sites where there is an indel gap in one sequence and not the other) was used to determine the gene clusters for family 4. R scripts are included as a supplementary file (S1 File).

### Genotyping pipeline

Our scripts and example datasets are available at: https://github.com/jyu429/IGHV-genotyping. We assume the WGS data is in BAM or SAM format [57], with reads already filtered to come from the IGHV locus. For WGS reads aligned to GRCh37, this is chr14:105,900,000-107,300,000. For reads aligned to GRCh38, this is chr14:105,700,000-106,900,000 (coordinates extend beyond the IGHV locus to be conservative). Bowtie2 [36] is used to map these reads to all functional, full-length IMGT alleles (the same set used for hierarchical clustering). The default Bowtie2 local alignment threshold led to too many multiple matches. S9 Fig illustrates how we increased this threshold to be more restrictive. Mapped reads are then pooled according to the gene clusters described in the Results section. For example, all reads that map to the alleles of segments 3-30, 3-30-3, 3-30-5, and 3-33 are pooled together. SPAdes *de novo* assembler [38] is run on the pooled reads for each operational segment. This assembler first performs error-correction on the reads and then attempts to piece together reads based on their overlap. SPAdes has an option to report diploid contigs (one for each chromosome), but running SPAdes with this option on the Platinum Genomes dataset did not produce more than one segment-length contig per gene cluster. The assembled contigs are compared with the IMGT database using stand-alone IgBLAST [39] to determine the closest matching allele, the length of match, and the number of nucleotide mutations or indels that separate the contig from the closest-matching allele. The read coverage depth of the contig as reported by SPAdes is also recorded for further analysis.

### Simulated reads

To test the capabilities and quality of our methods, ART [58] was used to generate simulated Illumina reads from GRCh37 and GRCh38 of lengths 70, 100, and 250 bp, each at coverage depths of 30×, 40×, and 50×. Error profiles of simulated reads and adjustments to default ART parameters are illustrated in S10 and S11 Figs.

### Filtering using mate-pair information

For the Platinum Genomes data, which comprises paired-end reads, we apply an additional filtering step to remove reads from pseudogenes that share a common subsequence with a functional gene. One way to identify reads of a pseudogene is to compare its mapped position with the position of its mate. If the mate read maps to a region that is substantially farther from the region the first read maps to (we use a threshold of 1000 bp to be conservative) then there is a chance it comes from a pseudogene and the original read is discarded. S12 Fig demonstrates that this filtering step eliminates more than half the reads from pseudogenes. Note that as a tradeoff, this filtering step will in some cases also incorrectly discard reads from duplicates that are located in a different region of the genome. For segments where the starting position relative to the genome is undetermined, no filtering occurs. In the case of the Platinum Genomes data, which is aligned to GRCH37, this means that filtering is not applied to reads from segments 7-4-1, 5-10-1, 4-38-2, 4-30-2, and 1-69-2. For gene clusters that comprise more than one V gene segment, we use the position of the first segment in the cluster (e.g. 3-53 for the gene cluster containing 3-53 and 3-66) as the mapped position of the first read. Depending on how uniquely mappable the segments within a cluster are, this can also result in underestimates of gene cluster copy number.

### Extra filtering step for novel 7-4-1 allele detection

Alleles of 7-4-1 have high nucleotide similarity to subsequences of pseudogenes 7-81, 7-40, and 7-34-1. The mate-pair filtering step above does not apply to 7-4-1 because the Platinum Genomes reads are aligned to GRCh37, which does not contain 7-4-1. To filter out reads from these pseudogenes for 7-4-1, we ran stand-alone IgBLAST on reads mapped to segment 7-4-1. The reads that had the highest match to a pseudogene were removed. The remaining reads were then used as input for SPAdes *de novo* assembler.

## Supporting Information

S1 Fig Hierarchical clustering applied to Hamming distance between all family 2 alleles. Heatmap color scale is same as in the main text, with red = 0% nucleotide differences, white = 10% or more.
(PDF)

S2 Fig Hierarchical clustering applied to Hamming distance between all family 5 alleles. Heatmap color scale is same as in the main text, with red = 0% nucleotide differences, white = 10% or more.
(PDF)

S3 Fig Hierarchical clustering applied to Hamming distance between all family 3 alleles. Allele labels are in cladogram above matrix. Heatmap color scale is same as in the main text, with red = 0% nucleotide differences, white = 10% or more.
(PDF)

S4 Fig Hierarchical clustering applied to all family 4 alleles. (A) Simple average of ‘TN93’ evolutionary distance and indel distance. (B) Hamming distance. Allele labels are in cladogram below matrix. Because TN93 and indel distances cannot be interpreted in terms of nucleotide similarity, the distances in each matrix have been normalized by the maximum value in the matrix for comparison. Heatmap color scale ranges from cyan = 0 to blue = 1. The clustering that uses ‘TN93’ and ‘indel’ distances has clearer block diagonal structure and fewer conflicts with IMGT nomenclature. It was therefore used to define the gene clusters for family 4 in Table 1.
(PDF)

S5 Fig Dotplots of estimated copy number for each individual in the Platinum Genomes dataset. The data points are the same as in Fig 4 but grouped by individuals rather than gene cluster. Y axis is normalized read coverage depth.
(PDF)

S6 Fig Estimated copy number of gene clusters in subjects NA12886 and NA12890. The absence of 1-8/3-9 and 5-10-1/3-64D variants does not appear to be due to VDJ recombination because positive copy number calls are made for gene clusters left of 1-8 and 3-9 (and toward the recombination site). Y axis is normalized read coverage depth.
(PDF)

S7 Fig Pairwise alignment of the putative 7-4-1 allele, 7-4-1*04_5, with its closest matching IMGT allele, 7-4-1*04. The allele 7-4-1*04_5 was found in individuals NA12877, NA12878, NA12879, NA12883, NA12884, NA12886, NA12888, NA12891, and NA12893. Pairwise alignment was performed using the online IgBLAST tool [39].
(PDF)

S8 Fig Allele calls may not reflect heterozygous state. Allele calls are arranged according to family pedigree. Only gene clusters for which there were two alleles in the family are shown (colored grey and white). Individuals for whom the gene cluster is not present are denoted by boxes with dashed outlines.
(PDF)

S9 Fig Mapped position versus original position of the start of each 250 bp read whose alignment exceeds the score threshold for segment 3-48. Axis values are centered at position chr14:1,062,766,005. (A) With default Bowtie2 local alignment threshold of 20 + 8.0 ln(*L*), where *L* is the read length, reads originally from pseudogenes or similar functional segments are incorrectly mapped to 3-48, as seen by multiple vertical strips of dots. (B) With the threshold increased to 20 + 70 ln(*L*), a single diagonal row of dots indicates that only reads from 3-48 are mapped to segment 3-48. (C) When the threshold is increased to 20 + 85 ln(*L*) however, this is too restrictive and too few reads are mapped. Assessing analogous plots for the rest of the segments led to a threshold of 20 + 70 ln(*L*) being chosen. The README of the package provides more detail on how to modify the threshold. (Coordinates are for chromosome 14 on GRCh37).
(PDF)

S10 Fig Error profiles of simulated reads under default ART parameters. Plots are shown for 30×, 40×, and 50× coverages and are only displayed for GRCh37 (plots for GRCh38 are similar). Note the high error rates for 100 bp and 70 bp reads. This difference is attributed to the fact that ART automatically selects one of several built-in read quality profiles according to the read length provided. Mutation rates are computed by first calculating, for each position in the read, the number of mismatches between the position of the simulated nucleotide and the original nucleotide. The number of mismatches was then divided by the total number of reads.
(PDF)

S11 Fig Error profiles of simulated reads after parameter adjustment. To make the profiles for 70 bp and 100 bp comparable to that of 250 bp, the parameter for quality score shifting (-qs and -qs2) was used: 12.896 for 100bp and 7.99 for 70bp.
(PDF)

S12 Fig Filtering using mate pair information decreases reads from pseudogenes. For each individual, we calculated the fraction of reads that more closely match a pseudogene than an IMGT allele (blue). We then did the same calculation after filtering reads using the location of the paired read (green).
(PDF)

S1 Appendix Read coverage adjustment when number of cells is known. (PDF)

S1 File R scripts for hierarchical clustering. (R)

S1 Table Simulated GRCh37 reads map ambiguously to IMGT alleles. Reads simulated from GRCh37 map to multiple IMGT alleles. Note that the in cases where reads map exactly to one allele, i.e. 3-72*01, 2-26*01, 1-24*01, and 3-20*01, these are the only full-length and functional alleles corresponding to a segment. Simulated reads are 100 bp long and have coverage depth of 30x.
(PDF)

S2 Table GRCh37 and GRCh38 in terms of our gene clusters. When there is one allele listed for a gene cluster, that gene cluster is considered to be in single copy. If there are two alleles listed, the gene cluster has two copies.
(PDF)

S3 Table Tab-separated (tsv) table of raw coverage depth calls from Platinum Genomes dataset. Data points in Fig 4 are obtained from this table as follows. For each individual, the raw coverage depth for each gene cluster has been divided by half of the coverage depth of segment 3-74 in that same individual. For example, the raw coverage for gene clusters in individual NA12877 are divided by half of 25.7855 (the coverage of 3-74). More details in the section ‘Genotyping the Platinum Genomes dataset’.
(TSV)
