# Supplementary material for: Estimating Copy Number and Allelic Variation at the Immunoglobulin Heavy Chain Locus Using Short Reads
Source: PLoS Comput Biol. 2016 Sep 15;12(9):e1005117. doi: 10.1371/journal.pcbi.1005117 (PMC5025152; doi:10.1371/journal.pcbi.1005117)
Supplement: S2 Fig — Heatmap color scale is same as in the main text, with red = 0% nucleotide differences, white = 10% or more. (PDF) [file pcbi.1005117.s002.pdf]

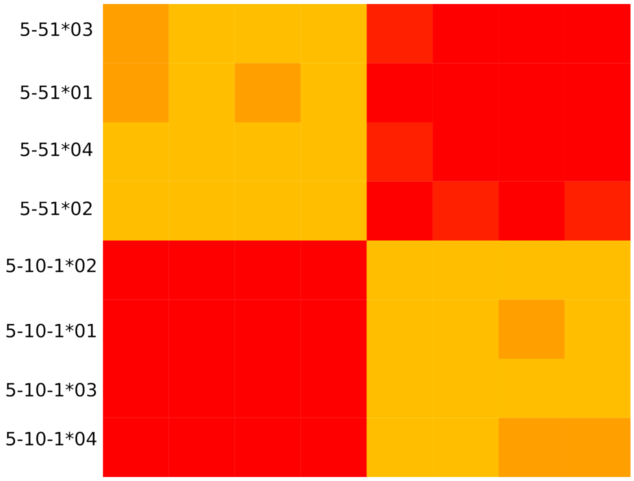

S2 Figure: Hierarchical clustering applied to Hamming distance between all family 5 alleles. Heatmap color scale is same as in the main text, with red=0% nucleotide differences, white=10% or more.
