# Supplementary material for: Estimating Copy Number and Allelic Variation at the Immunoglobulin Heavy Chain Locus Using Short Reads
Source: PLoS Comput Biol. 2016 Sep 15;12(9):e1005117. doi: 10.1371/journal.pcbi.1005117 (PMC5025152; doi:10.1371/journal.pcbi.1005117)
Supplement: S5 Fig — The data points are the same as in Fig 4 but grouped by individuals rather than gene cluster. Y axis is normalized read coverage depth. (PDF) [file pcbi.1005117.s005.pdf]

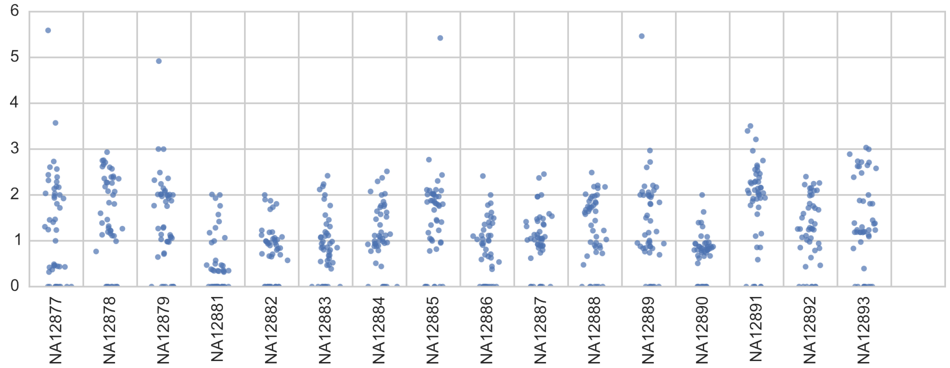

S5 Figure: Dotplots of estimated copy number for each individual in the Platinum Genomes dataset. The data points are the same as in Fig. 4 but grouped by individuals rather than gene cluster. Y axis is normalized read coverage depth.
