# Supplementary material for: Estimating Copy Number and Allelic Variation at the Immunoglobulin Heavy Chain Locus Using Short Reads
Source: PLoS Comput Biol. 2016 Sep 15;12(9):e1005117. doi: 10.1371/journal.pcbi.1005117 (PMC5025152; doi:10.1371/journal.pcbi.1005117)
Supplement: S6 Fig — The absence of 1-8/3-9 and 5-10-1/3-64D variants does not appear to be due to VDJ recombination because positive copy number calls are made for gene clusters left of 1-8 and 3-9 (and toward the recombination site). Y axis is normalized read coverage depth. (PDF) [file pcbi.1005117.s006.pdf]

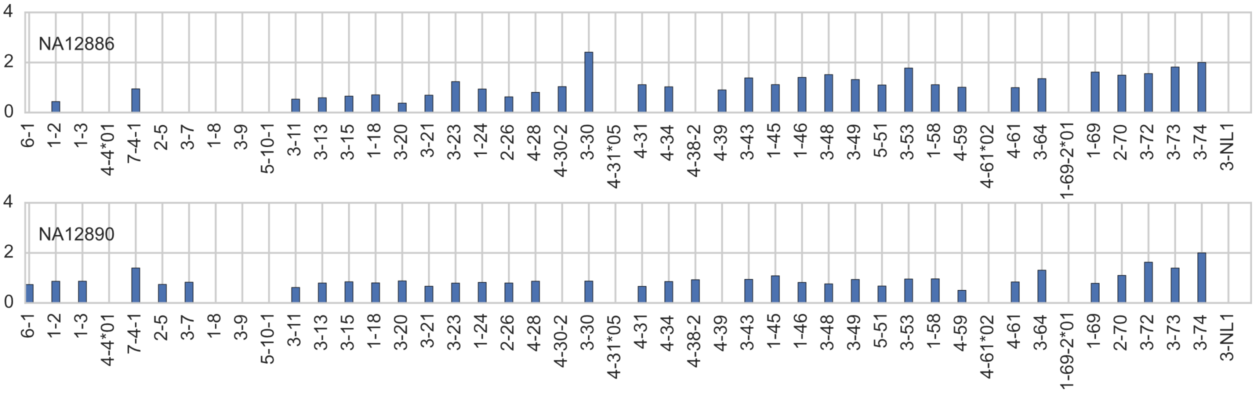

S6 Figure: Estimated copy number of gene clusters in subjects NA12886 and NA12890. The absence of 1-8/3-9 and 5-10-1/3-64D variants does not appear to be due to VDJ recombination because positive copy number calls are made for gene clusters left of 1-8 and 3-9 (and toward the recombination site). Y axis is normalized read coverage depth.
