# Supplementary material for: Estimating Copy Number and Allelic Variation at the Immunoglobulin Heavy Chain Locus Using Short Reads
Source: PLoS Comput Biol. 2016 Sep 15;12(9):e1005117. doi: 10.1371/journal.pcbi.1005117 (PMC5025152; doi:10.1371/journal.pcbi.1005117)
Supplement: S7 Fig — The allele 7-4-1*04_5 was found in individuals NA12877, NA12878, NA12879, NA12883, NA12884, NA12886, NA12888, NA12891, and NA12893. Pairwise alignment was performed using the online IgBLAST tool [39]. (PDF) [file pcbi.1005117.s007.pdf]

```
                    <-------------------------------FR1-IMGT-----------------------------------><-------CDR1-IM
                      Q  V  Q  L  V  Q  S  G  S  E  L  K  K  P  G  A  S  V  K  V  S  C  K  A  S  G  Y  T  F  T
7-4-1*04_5       1   CAGGTGCAGCTGGTGCAATCTGGGTCTGAGTTGAAGAAGCCTGGGGCCTCAGTGAAGGTTTCCTGCAAGGCTTCTGGATACACCTTCACT  90
7-4-1*04         1   ..........................................................................................  90
                      Q  V  Q  L  V  Q  S  G  S  E  L  K  K  P  G  A  S  V  K  V  S  C  K  A  S  G  Y  T  F  T

                    GT------><--------------------FR2-IMGT----------------------><-------CDR2-IMGT------><-----
                      S  Y  A  M  N  W  V  R  Q  A  P  G  Q  G  L  E  W  M  G  W  I  N  T  N  T  G  N  L  T  Y
7-4-1*04_5       91  AGCTATGCTATGAATTGGGTGCGACAGGCCCCTGGACAAGGGCTTGAGTGGATGGGATGGATCAACACCAACACTGGGAACCTAACGTAT  180
7-4-1*04         91  ..................................................................................C.......  180
                      S  Y  A  M  N  W  V  R  Q  A  P  G  Q  G  L  E  W  M  G  W  I  N  T  N  T  G  N  P  T  Y

                    -----------------------------------------------FR3-IMGT------------------------------------
                      A  Q  G  F  T  G  R  F  V  F  S  M  D  T  S  V  S  M  A  Y  L  H  I  S  S  L  K  A  E  D
7-4-1*04_5       181 GCCCAGGGCTTCACAGGACGGTTTGTCTTCTCCATGGACACCTCCGTCAGCATGGCATATCTTCATATCAGCAGCCTAAAGGCTGAGGAC  270
7-4-1*04         181 .................................T..........T.................G..G........................  270
                      A  Q  G  F  T  G  R  F  V  F  S  L  D  T  S  V  S  M  A  Y  L  Q  I  S  S  L  K  A  E  D

                    ----------------->
                      T  A  V  Y  Y  C  A  R
7-4-1*04_5       271 ACTGCCGTGTATTACTGTGCGAGAGA  296
7-4-1*04         271 ..........................  296
                      T  A  V  Y  Y  C  A  R
```

S7 Figure: Pairwise alignment of the putative 7-4-1 allele, 7-4-1*04_5, with its closest matching IMGT allele, 7-4-1*04. The allele 7-4-1*04_5 was found in individuals NA12877, NA12878, NA12879, NA12883, NA12884, NA12886, NA12888, NA12891, and NA12893. Pairwise alignment was performed using the online IgBLAST tool.
