# Supplementary material for: Estimating Copy Number and Allelic Variation at the Immunoglobulin Heavy Chain Locus Using Short Reads
Source: PLoS Comput Biol. 2016 Sep 15;12(9):e1005117. doi: 10.1371/journal.pcbi.1005117 (PMC5025152; doi:10.1371/journal.pcbi.1005117)
Supplement: S8 Fig — Allele calls are arranged according to family pedigree. Only gene clusters for which there were two alleles in the family are shown (colored grey and white). Individuals for whom the gene cluster is not present are denoted by boxes with dashed outlines. (PDF) [file pcbi.1005117.s008.pdf]

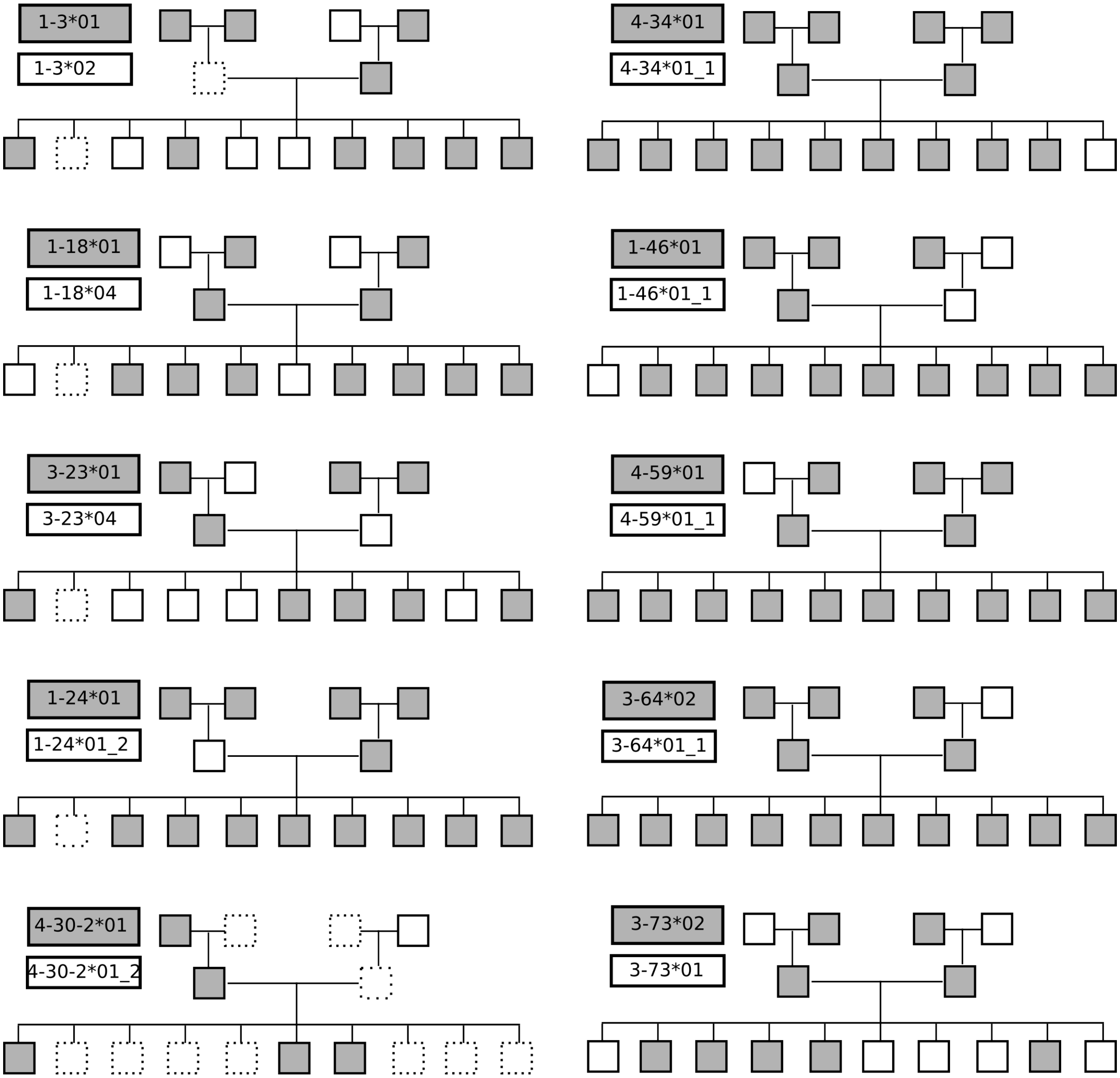

S8 Figure: Allele calls may not reflect heterozygous genes. Allele calls are arranged according to family pedigree. Only gene clusters for which there were two alleles in the family are shown (colored grey and white). Individuals for whom the gene cluster is not present are denoted by boxes with dashed outlines.
