# Supplementary material for: Estimating Copy Number and Allelic Variation at the Immunoglobulin Heavy Chain Locus Using Short Reads
Source: PLoS Comput Biol. 2016 Sep 15;12(9):e1005117. doi: 10.1371/journal.pcbi.1005117 (PMC5025152; doi:10.1371/journal.pcbi.1005117)
Supplement: S9 Fig — Axis values are centered at position chr14:1,062,766,005. (A) With default Bowtie2 local alignment threshold of 20 + 8.0 ln(L), where L is the read length, reads originally from pseudogenes or similar functional segments are incorrectly mapped to 3-48, as seen by multiple vertical strips of dots. (B) With the threshold increased to 20 + 70 ln(L), a single diagonal row of dots indicates that only reads from 3-48 are mapped to segment 3-48. (C) When the threshold is increased to 20 + 85 ln(L) however, this is too restrictive and too few reads are mapped. Assessing analogous plots for the rest of the segments led to a threshold of 20 + 70 ln(L) being chosen. The README of the package provides more detail on how to modify the threshold. (Coordinates are for chromosome 14 on GRCh37). (PDF) [file pcbi.1005117.s009.pdf]

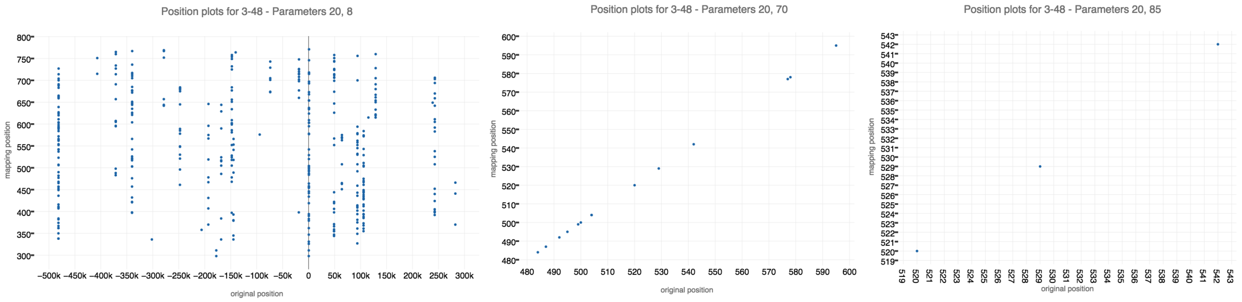

S9 Figure: Mapped position versus original position of the start of each 250 bp read whose alignment exceeds the score threshold for segment 3-48. Axis values are centered at position chr14:1,062,766,005. (A) With default Bowtie2 local alignment threshold of $20 + 8.0 \ln(L)$, where L is the read length, reads originally from pseudogenes or similar functional segments are incorrectly mapped to 3-48, as seen by multiple vertical strips of dots. (B) With the threshold increased to $20 + 70 \ln(L)$, a single diagonal row of dots indicates that only reads from 3-48 are mapped to segment 3-48. (C) When the threshold is increased to $20 + 85 \ln(L)$ however, this is too restrictive and too few reads are mapped. Assessing analogous plots for the rest of the segments led to a threshold of $20 + 70 \ln(L)$ being chosen. The README of the package provides more detail on how to modify the threshold. (Coordinates are for chromosome 14 on GRCh37).
