# Supplementary material for: Estimating Copy Number and Allelic Variation at the Immunoglobulin Heavy Chain Locus Using Short Reads
Source: PLoS Comput Biol. 2016 Sep 15;12(9):e1005117. doi: 10.1371/journal.pcbi.1005117 (PMC5025152; doi:10.1371/journal.pcbi.1005117)
Supplement: S10 Fig — Plots are shown for 30×, 40×, and 50× coverages and are only displayed for GRCh37 (plots for GRCh38 are similar). Note the high error rates for 100 bp and 70 bp reads. This difference is attributed to the fact that ART automatically selects one of several built-in read quality profiles according to the read length provided. Mutation rates are computed by first calculating, for each position in the read, the number of mismatches between the position of the simulated nucleotide and the original nucleotide. The number of mismatches was then divided by the total number of reads. (PDF) [file pcbi.1005117.s010.pdf]

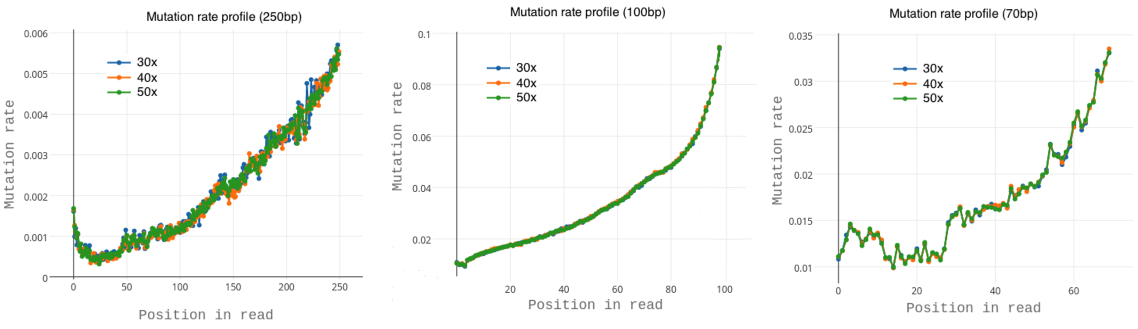

S10 Figure: Error profiles of simulated reads under default ART parameters. Plots are shown for 30x, 40x, and 50x coverages and are only displayed for GRCh37 (plots for GRCh38 are similar). Note the high error rates for 100 bp and 70 bp reads. This difference is attributed to the fact that ART automatically selects one of several built-in read quality profiles according to the read length provided. Mutation rates are computed by first calculating, for each position in the read, the number of mismatches between the position of the simulated nucleotide and the original nucleotide. The number of mismatches was then divided by the total number of reads.
