# Supplementary material for: Estimating Copy Number and Allelic Variation at the Immunoglobulin Heavy Chain Locus Using Short Reads
Source: PLoS Comput Biol. 2016 Sep 15;12(9):e1005117. doi: 10.1371/journal.pcbi.1005117 (PMC5025152; doi:10.1371/journal.pcbi.1005117)
Supplement: S11 Fig — To make the profiles for 70 bp and 100 bp comparable to that of 250 bp, the parameter for quality score shifting (-qs and -qs2) was used: 12.896 for 100bp and 7.99 for 70bp. (PDF) [file pcbi.1005117.s011.pdf]

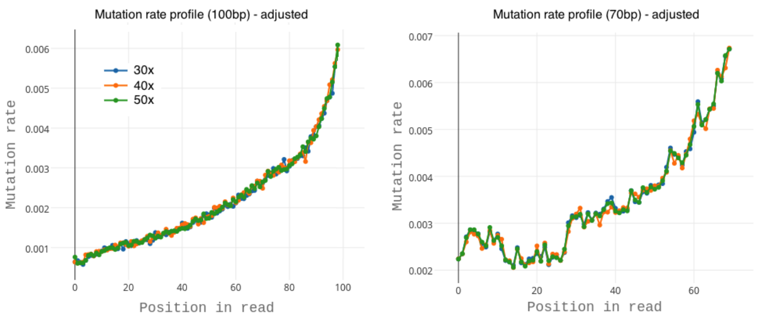

S11 Figure: Error profiles of simulated reads after parameter adjustment. To make the profiles for 70 bp and 100 bp comparable to that of 250 bp, the parameter for quality score shifting (-qs and -qs2) was used: 12.896 for 100bp and 7.99 for 70bp.
