# Supplementary material for: Estimating Copy Number and Allelic Variation at the Immunoglobulin Heavy Chain Locus Using Short Reads
Source: PLoS Comput Biol. 2016 Sep 15;12(9):e1005117. doi: 10.1371/journal.pcbi.1005117 (PMC5025152; doi:10.1371/journal.pcbi.1005117)
Supplement: S12 Fig — For each individual, we calculated the fraction of reads that more closely match a pseudogene than an IMGT allele (blue). We then did the same calculation after filtering reads using the location of the paired read (green). (PDF) [file pcbi.1005117.s012.pdf]

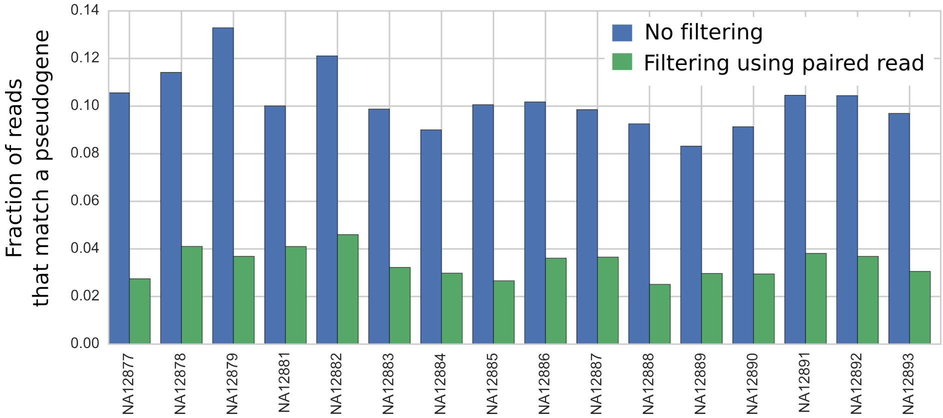

S12 Figure: Filtering using mate pair information decreases reads from pseudogenes. For each individual, we calculated the fraction of reads that more closely match a pseudogene than an IMGT allele (blue). We then did the same calculation after filtering reads using the location of the paired read (green).
