# Supplementary material for: Estimating Copy Number and Allelic Variation at the Immunoglobulin Heavy Chain Locus Using Short Reads
Source: PLoS Comput Biol. 2016 Sep 15;12(9):e1005117. doi: 10.1371/journal.pcbi.1005117 (PMC5025152; doi:10.1371/journal.pcbi.1005117)
Supplement: S1 Appendix — (PDF) [file pcbi.1005117.s013.pdf]

S1 Appendix: Read coverage adjustment when number of cells is known

Assume that the 6-1 and 3-74 segments are at opposite ends of the IGHV locus (with 6-1 at the centromeric end) and that both are present as single copies on each chromosome. This assumption is reasonable because there are no documented examples of copy number variation in these two segments, they are at the ends for both known reference genomes, and they each only have two allelic variants.

Reads from 6-1 can only appear in an immortalized B lymphocyte sample if they come from a non-rearranged haplotype or a rearranged haplotype with 6-1 as the V segment that underwent rearrangement. We ignore the latter possibility for now because assuming rearrangement is uniform over the V segments, the probability 6-1 is involved in a rearrangement is about 1/40 or 0.025, and we are unlikely to obtain such precision in our estimate for the fraction of rearranged haplotypes in the sample for this amount to matter.

One point estimate for the fraction of haplotypes in a sample that are rearranged is thus

$$p_{\text{VDJ}} \approx 1 - \frac{\text{number reads from 6-1}}{\text{number reads from 3-74}}.$$

Now suppose there are m segments in total and we are interested in how the observed read count of segment i (ith counting from the 6-1 end) is related to the true abundance of segment i. The reads we observe in the data will be a mixture of reads from non-rearranged and rearranged IGHV loci. The non-rearranged loci are expected to contribute reads in proportion to the true abundance of the segment. The rearranged loci will only contribute reads if the breakpoint of the rearrangement does not include segment i. Let us assume for now that the breakpoints occur uniformly over the locus so that this event occurs with probability $\frac{i}{m}$ (we show how to relax this assumption later). Thus,

$$\begin{aligned}
&\mathbb{E}(\text{observed number reads for segment } i) \\
&\propto (1 - p_{\text{VDJ}}) \cdot \text{true abundance of } i + p_{\text{VDJ}} \cdot \text{true abundance of } i \cdot \frac{i}{m} \\
&= \text{true abundance of } i \cdot \left[1 - p_{\text{VDJ}}\left(1 - \frac{i}{m}\right)\right]
\end{aligned}$$

Thus, we may estimate the true abundance of segment i with

$$\frac{\text{observed number reads for segment } i}{\left[1 - p_{\text{VDJ}}\left(1 - \frac{i}{m}\right)\right]}.$$

This is only a point estimate however, and without knowing the number of haplotypes in the sample (number of B cells that were sequenced), it is not possible to quantify the uncertainty of this estimate.

Allowing for non-uniform VDJ breakpoints

In the above, we assume V segments are sampled uniformly with the same probability, $\frac{1}{m}$, during VDJ rearrangement. Suppose, instead, that segment i has probability f_i of being sampled.

The probability that a VDJ rearrangement does not include segment i is now $\sum_{h=1}^{i} f_h$. Thus, we may estimate the true abundance of segment i with

$$\frac{\text{observed number reads for segment } i}{\left[1 - p_{\text{VDJ}}\left(1 - \sum_{h=1}^{i} f_h\right)\right]}.$$

To compare this with our earlier result with uniform VDJ recombinants, set $f_i = \frac{1}{m}$ and note that $\sum_{h=1}^{i} f_h = \sum_{h=1}^{i} \frac{1}{m} = \frac{i}{m}$.
