# Supplementary material for: Estimating Copy Number and Allelic Variation at the Immunoglobulin Heavy Chain Locus Using Short Reads
Source: PLoS Comput Biol. 2016 Sep 15;12(9):e1005117. doi: 10.1371/journal.pcbi.1005117 (PMC5025152; doi:10.1371/journal.pcbi.1005117)
Supplement: S1 Table — Reads simulated from GRCh37 map to multiple IMGT alleles. Note that the in cases where reads map exactly to one allele, i.e. 3-72*01, 2-26*01, 1-24*01, and 3-20*01, these are the only full-length and functional alleles corresponding to a segment. Simulated reads are 100 bp long and have coverage depth of 30x. (PDF) [file pcbi.1005117.s015.pdf]

Table S1: Simulated GRCh37 reads map ambiguously to IMGT alleles. Reads simulated from GRCh37 map to multiple IMGT alleles. Note that the in cases where reads map exactly to one allele, i.e. 3-72*01, 2-26*01, 1-24*01, and 3-20*01, these are the only full-length and functional alleles corresponding to a segment. Simulated reads are 100 bp long and have coverage depth of 30x.

Read from	Is mapped to
3-74*01	3-74*01, 3-74*02, 3-74*03
3-73*02	3-73*01, 3-73*02
3-72*01	3-72*01
2-70*13	2-70*01, 2-70*10, 2-70*11, 2-70*12, 2-70*13
1-69*06	1-69*01, 1-69*02, 1-69*04, 1-69*05, 1-69*06, 1-69*08, 1-69*09, 1-69*12, 1-69*14
3-66*03	3-30*14, 3-53*01, 3-53*04, 3-66*02, 3-66*03
3-64*02	3-64*01, 3-64*02
4-61*08	4-59*01, 4-59*02, 4-59*03, 4-59*07, 4-61*01, 4-61*03, 4-61*08
4-59*01	4-4*08, 4-59*01, 4-59*02, 4-59*03, 4-59*04, 4-59*07, 4-59*08, 4-61*01, 4-61*03, 4-61*05, 4-61*08
1-58*02	1-58*01, 1-58*02
3-53*01	3-53*01, 3-53*02, 3-53*03, 3-53*04, 3-66*02, 3-66*03
5-51*01	5-51*01, 5-51*02, 5-51*03, 5-51*04
3-49*03	3-49*01, 3-49*02, 3-49*03, 3-49*04, 3-49*05
3-48*02	3-13*01, 3-13*04, 3-23*04, 3-48*01, 3-48*02, 3-48*04
1-46*01	1-46*01, 1-46*02, 1-46*03, 3-11*04, 3-11*06, 3-48*03, 3-48*04, 3-66*01, 3-7*01, 3-7*02, 3-7*03
1-45*02	1-45*01, 1-45*02
3-43*01	3-43*01, 3-43*02, 3-43D*01
4-39*01	4-30-2*03, 4-39*01, 4-39*02, 4-39*05, 4-39*06, 4-39*07, 4-59*05, 4-61*05
4-34*01	4-34*01, 4-34*02, 4-34*04, 4-34*05, 4-34*08, 4-34*12
3-33*01	3-30*02, 3-30*06, 3-30*07, 3-30*11, 3-30*12, 3-30-3*02, 3-33*01, 3-33*02, 3-33*03, 3-33*04, 3-33*05, 3-33*06
4-31*02	4-30-4*01, 4-31*01, 4-31*02, 4-31*03, 4-31*04, 4-31*05, 4-59*06
3-30*03	3-30*02, 3-30*03, 3-30*04, 3-30*05, 3-30*06, 3-30*07, 3-30*10, 3-30*13, 3-30*17, 3-30*18, 3-30-3*02, 3-30-3*03, 3-33*01, 3-33*05
4-28*01	4-28*01, 4-28*02, 4-28*03, 4-28*04, 4-28*05, 4-28*07
2-26*01	2-26*01
1-24*01	1-24*01
3-23*01	3-23*01, 3-23*02, 3-23*03, 3-23*04, 3-23D*01
3-21*01	3-11*06, 3-21*01, 3-21*02, 3-21*03, 3-21*04
3-20*01	3-20*01
1-18*01	1-18*01, 1-18*03, 1-18*04
3-15*01	3-15*01, 3-15*02, 3-15*04, 3-15*05, 3-15*06, 3-15*07
3-13*01	3-13*01, 3-13*03, 3-13*04, 3-13*05, 3-23*04, 3-48*02, 3-7*01, 3-7*02, 3-7*03
3-11*01	3-11*01, 3-11*03, 3-11*04, 3-11*05, 3-11*06
3-9*01	3-9*01, 3-9*02, 3-9*03
1-8*01	1-2*01, 1-2*02, 1-2*04, 1-8*01, 1-8*02
3-7*01	1-46*01, 1-46*02, 1-46*03, 3-21*01, 3-21*02, 3-64*01, 3-7*01, 3-7*02, 3-7*03
2-5*01	2-5*01, 2-5*02, 2-5*04, 2-5*05, 2-5*08, 2-5*09
4-4*07	4-39*07, 4-4*07, 4-4*08, 4-59*03, 4-59*04, 4-59*10, 4-61*02
1-3*02	1-3*01, 1-3*02
1-2*02	1-2*01, 1-2*02, 1-2*03, 1-2*04, 1-2*05
6-1*01	6-1*01, 6-1*02
