# Supplementary material for: Estimating Copy Number and Allelic Variation at the Immunoglobulin Heavy Chain Locus Using Short Reads
Source: PLoS Comput Biol. 2016 Sep 15;12(9):e1005117. doi: 10.1371/journal.pcbi.1005117 (PMC5025152; doi:10.1371/journal.pcbi.1005117)
Supplement: S2 Table — When there is one allele listed for a gene cluster, that gene cluster is considered to be in single copy. If there are two alleles listed, the gene cluster has two copies. (PDF) [file pcbi.1005117.s016.pdf]

Table S2: GRCh37 and GRCh38 in terms of our gene clusters. When there is one allele listed for a gene cluster, that gene cluster is considered to be in single copy. If there are two alleles listed, the gene cluster has two copies.

Gene cluster	GRCh37	GRCh38
6-1	6-1*01	6-1*01
1-2	1-2*02	1-2*04
1-3	1-3*02	1-3*01
4-4*01	-	4-4*02
7-4-1		7-4-1*01
2-5	2-5*01	2-5*02
3-7	3-7*01	3-7*03
1-8	1-8*01	-
3-9	3-9*01	-
5-10-1	-	5-10-1*03
3-11	3-11*01	3-11*06
3-13	3-13*01	3-13*05
3-15	3-15*01	3-15*01
1-18	1-18*01	1-18*04
3-20	3-20*01	3-20*02
3-21	3-21*01	3-21*01
3-23	3-23*01	3-23*04
1-24	1-24*01	1-24*01
2-26	2-26*01	2-26*01
4-28	4-28*01	4-28*07
4-30-2	-	4-30-2*01
3-30	3-33*01, 3-30*03	3-30*18, 3-33*01
4-31	4-31*02	-
4-34	4-34*01	4-34*01
4-39	4-39*01	4-39*01
3-43	3-43*01	3-43*01
1-45	1-45*02	1-45*02
1-46	1-46*01	1-46*01
3-48	3-48*02	3-48*03
3-49	3-49*03	3-49*04
5-51	5-51*01	5-51*01
3-53	3-53*01, 3-66*03	3-53*02, 3-66*03
1-58	1-58*02	1-58*01
4-59	4-4*07, 4-59*01	4-59*01
4-61	4-61*08	4-61*01
3-64	3-64*02	3-64*02, 3-64D*06
1-69-2	-	1-69-2*01
1-69	1-69*06	1-69D*01, 1-69*06
2-70	2-70*13	2-70D*04, 2-70*01
3-72	3-72*01	3-72*01
3-73	3-73*02	3-73*02
3-74	3-74*01	3-74*01
