# Supplementary material for: Estimating Copy Number and Allelic Variation at the Immunoglobulin Heavy Chain Locus Using Short Reads
Source: PLoS Comput Biol. 2016 Sep 15;12(9):e1005117. doi: 10.1371/journal.pcbi.1005117 (PMC5025152; doi:10.1371/journal.pcbi.1005117)
Supplement: S4 Fig — (A) Simple average of ‘TN93’ evolutionary distance and indel distance. (B) Hamming distance. Allele labels are in cladogram below matrix. Because TN93 and indel distances cannot be interpreted in terms of nucleotide similarity, the distances in each matrix have been normalized by the maximum value in the matrix for comparison. Heatmap color scale ranges from cyan = 0 to blue = 1. The clustering that uses ‘TN93’ and ‘indel’ distances has clearer block diagonal structure and fewer conflicts with IMGT nomenclature. It was therefore used to define the gene clusters for family 4 in Table 1. (PDF) [file pcbi.1005117.s004.pdf]

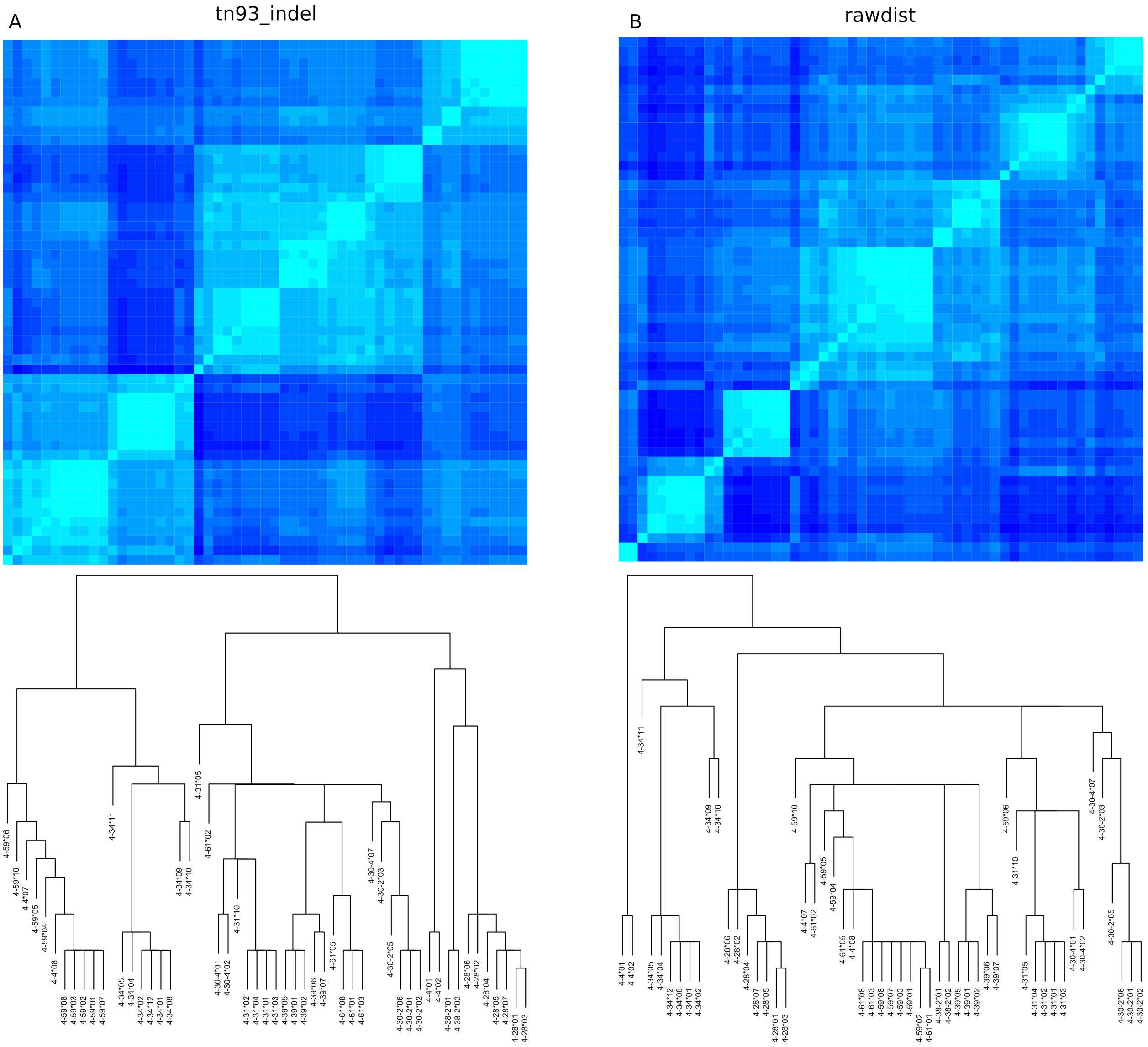


**S4 Figure: Hierarchical clustering applied to all family 4 alleles.** (A) Simple average of 'TN93' evolutionary distance and indel distance. (B) Hamming distance. Allele labels are in cladogram below matrix. Because TN93 and indel distances cannot be interpreted in terms of nucleotide similarity, the distances in each matrix have been normalized by the maximum value in the matrix for comparison. Heatmap color scale is cyan=0 and blue=1. The clustering that uses 'TN93' and 'indel' distances has clearer block diagonal structure and fewer conflicts with IMGT nomenclature. It was therefore used to define the gene clusters for family 4 in Table 1.
